# Comparison of Machine Learning Methods for the Arterial Hypertension Diagnostics

**DOI:** 10.1155/2017/5985479

**Published:** 2017-07-31

**Authors:** Vladimir S. Kublanov, Anton Yu. Dolganov, David Belo, Hugo Gamboa

**Affiliations:** ^1^Research Medical and Biological Engineering Centre of High Technologies, Ural Federal University, Mira 19, Yekaterinburg 620002, Russia; ^2^Laboratório de Instrumentação, Engenharia Biomédica e Física da Radiação (LIBPhys-UNL), Departamento de Física, Faculdade de Ciências e Tecnologia da Universidade Nova de Lisboa, Monte da Caparica, 2892-516 Caparica, Portugal

## Abstract

The paper presents results of machine learning approach accuracy applied analysis of cardiac activity. The study evaluates the diagnostics possibilities of the arterial hypertension by means of the short-term heart rate variability signals. Two groups were studied: 30 relatively healthy volunteers and 40 patients suffering from the arterial hypertension of II-III degree. The following machine learning approaches were studied: linear and quadratic discriminant analysis, *k*-nearest neighbors, support vector machine with radial basis, decision trees, and naive Bayes classifier. Moreover, in the study, different methods of feature extraction are analyzed: statistical, spectral, wavelet, and multifractal. All in all, 53 features were investigated. Investigation results show that discriminant analysis achieves the highest classification accuracy. The suggested approach of noncorrelated feature set search achieved higher results than data set based on the principal components.

## 1. Introduction

According to the World Health data, hypertension affects more than 1 billion people worldwide. Many factors can conduce to hypertension, including occipital stress and job strain [1]. One of the main problems concerning the treatments for the arterial hypertension conditions is the late detection for apparently healthy people. Some studies had been shown that among the individuals with hypertension, more than 35% were unaware of their condition [2].

The heart rate variability (HRV) is among one of the widely used biomedical signals, due to ease of record the electrical heart activity [3]. The HRV analysis can be applied in the task of arterial hypertension diagnostics, since it is well known that various features of the HRV reflect behavior of the different modules of the autonomic nervous system (ANS) [4].

Common HRV analysis implies the application of a variety of analysis methods: statistical, spectral, and nonlinear analysis. Generally, in a single study, a limited number of features are extracted. Such as in [5], 13 nonlinear features were studied for efficacy of stress state detection. The current paper comprises a study of 53 different features. Usually during one study, feature sets of a particular method are used. For example in [6], sets of time-domain features, nonlinear features, and spectral features were studied separately for automatic sleep staging by means of HRV signal analyses. In this study, combinations of different methods were analyzed.

The common uses of machine learning approaches for condition classification based on HRV information imply usage of several available methods: support vector machine (SVM), discriminant analysis (DA), and ordinal pattern statistics (OPS) [7–9]. However, the selection of a particular approach is not always justified. In current study, linear and quadratic DA, SVM, *k*-nearest neighbors, decision trees, and Naïve Bayes approaches were investigated.

In one of the previous works, the investigation of the linear and quadratic discriminant analysis was carried out, implying the study of arterial hypertension diagnostic using single features of short-term HRV signals. In that work, the evaluation of the features and the evaluation of the classifier efficacy were carried out by means of an in-house software produced in MATLAB [10]. In the present paper, the machine learning methods were implemented in the python.

In summary, the goal of the present work is to study the efficacy of different machine learning approaches for diagnostic of the arterial hypertension by means of the short-term HRV, using combinations of statistical, spectral (Fourier and Wavelet transforms), and nonlinear features. By applying feature combinations of different methods, we aim to build more robust and accurate classifiers.

## 2. Materials and Methods

### 2.1. Recorded Dataset

The clinical part of the study was performed in the Sverdlovsk Clinical Hospital of Mental Diseases for Military Veterans (Yekaterinburg, Russian Federation). For the HR signals registration, the electroencephalograph-analyzer “Encephalan-131-03” (“Medicom-MTD,” Taganrog, Russian Federation) was used. The rotating table Lojer (Vammalan Konepaja OY, Finland) performed the spatial position change of the patient during passive orthostatic load; the lift of the head end of the table was up to 70° from the horizontal position. The clinical part of the study was approved by the local Ethics Committee of the Ural State Medical University.

**Figure 1:**
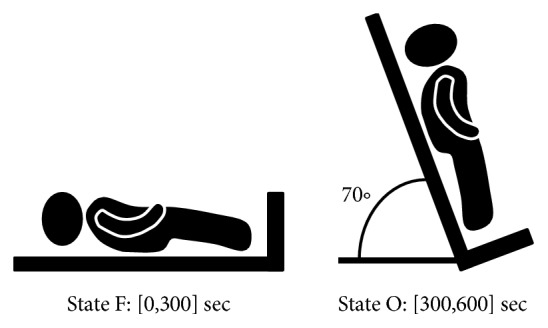
Diagram of the study.

Participants of this study were 30 healthy volunteers and 41 patients suffering from the arterial hypertension of II and III degree. The electrocardiography (ECG) signals were recorded in two functional states: functional rest (state F) and passive orthostatic load (state O). The length of the signal in the mentioned state was about 300 seconds. The HRV signals were consequently derived from the ECG signals automatically by the “Encephalan-131-03” software.  Figure 1 presents diagram of the functional states.

### 2.2. Heart Rate Variability Features

Prior to the processing, the original time series were cleaned from the artifacts. By artifacts, in this study, we considered values of the R-R intervals that differed from the HR mean by more than three standard deviations. NN is the abbreviation for the “normal to normal” time series, that is, without artifacts. Among all studied time series, less than 2% of data was removed. For spectral and multifractal analyses, NN time series were interpolated using cubic spline interpolation with the 10 Hz sampling frequency.

The feature dataset is the same as used previously [11], where it was shown that features of the HRV signals recorded in the state O have better classification accuracy for arterial hypertension diagnostics. Therefore, in this study, we analyze data only in the state O. The used features were separated into statistical, geometric, spectral (Fourier based), wavelet, nonlinear, and multifractal. Their description will be given below.

#### 2.2.1. Statistical Features

Statistical methods are used for the direct quantitative evaluation of the HR time series. Main quantitative features are as follows:

(i) *M* is the mean value of the R-R intervals after artifact rejection:
  (1) $$\begin{array}{c} M=\frac{1}{N}\overset{N}{\underset{i=1}{\sum }}{NN}_{i}, \end{array}$$
  where *N* is the number of elements in the NN and NN_*i*_ is the *i*th element in R-R time series.
(ii) HR, the heart rate, is an inverse ratio to *M*:
  (2) $$\begin{array}{c} HR=\frac{1}{M}. \end{array}$$
(iii) SDNN is the standard deviation of the NN intervals:
  (3) $$\begin{array}{c} SDNN=\sqrt{\frac{1}{N}-1\overset{N}{\underset{i=1}{\sum }}{\left({NN}_{i}-M\right)}^{2}}. \end{array}$$
(iv) CV, the coefficient of variation, is defined as ratio of standard deviation SDNN to the mean *M*, expressed in percent;
  (4) $$\begin{array}{c} CV=\frac{SDNN}{M}\cdot 100\%. \end{array}$$
(v) RMSSD is the square root of mean of squares of differences between successive elements in NN;
  (5) $$\begin{array}{c} RMSDD={\left[\frac{1}{N}\overset{N-1}{\underset{i=1}{\sum }}{\left({NN}_{i+1}-{NN}_{i}\right)}^{2}\right]}^{0,5}. \end{array}$$
(vi) NN50 is the number of pairs of successive elements in NN that differ by more than 50 ms [12].

#### 2.2.2. Geometric Features

The geometric methods analyze the distribution of the R-R intervals as a random numbers. The common features of these methods are as follows:

- *M*_0_, the mode, is the most frequent value in the R-R interval. In case of the normal distribution, the mode is close to the mean *M*.
- VR, the variation range, is the difference between the lowest R-R interval and the highest R-R interval in the time series. VR shows variability of the R-R interval values and reflects activity of the parasympathetic system of the ANS.
- AM_0_, the amplitude of the mode, is a number of the R-R intervals that correspond to the mode value. AM_0_ shows the stabilizing effect of the heart rate management, mainly caused by the sympathetic activity [12].

The following indexes are derived from common geometric features:

(i) SI, the stress index, reflects centralization degree of the heart rate and mostly characterizes the activity of the sympathetic department of the ANS:
  (6) $$\begin{array}{c} SI=\frac{{AM}_{0}}{2M_{0}\cdot VR}. \end{array}$$
(ii) IAB, the index of the autonomic balance, depends on the relation between activities of the sympathetic and parasympathetic department of the ANS:
  (7) $$\begin{array}{c} IAB=\frac{{AM}_{0}}{VR}. \end{array}$$
(iii) ARI, the autonomic rhythm index, shows parasympathetic shifts of the autonomic balance: smaller values of the ARI correspond to the shift of the autonomic balance to the parasympathetic activity:
  (8) $$\begin{array}{c} ARI=\frac{1}{M_{0}\cdot VR}. \end{array}$$
(iv) IARP, the index of adequate regulation processes, reflects accordance of the autonomic function changes of the sinus node as a reaction of the sympathetic regulatory effects on the heart:
  (9) $$\begin{array}{c} IARP=\frac{{AM}_{0}}{M_{0}}. \end{array}$$

#### 2.2.3. Spectral Features

Spectral analysis is used to quantify periodic processes in the heart rate by the means of the Fourier transform (Fr). The main spectral components of the HRV signal are high frequency—HF (0.4–0.15 Hz), low frequency—LF (0.15–0.04 Hz), very low frequency—VLF (0.04–0.003 Hz), and ultralow frequency—ULF (lower than 0.003 Hz) [12, 13]. For smaller than 300 seconds, short-term time series ULF spectral component is not analyzed.

HF spectral component characterizes activity of the parasympathetic system of the ANS and activity of the autonomic regulation loop. High frequencies of the heart rate in HRV spectrum are associated with the breathing and determined by the connection and influences of the vagus with the sinus node.

LF spectral component mainly characterizes activity of the sympathetic vascular tone regulation center. Low frequencies reflect modulation of the heart rate by the sympathetic nervous system [4].

VLF spectral component is defined by the suprasegmental regulation of the heart rate, as the amplitude of the VLF waves and is related to the psycho-emotional strain and functional state of the cortex. The genesis of the very low frequencies is still the matter of debates. Most likely, it is influenced by the suprasegmental centers of the autonomic regulation that generates slow rhythms. These rhythms are directed to the heart by the sympathetic nervous system, humoral factors on the sinus node. Biologic rhythms in the same frequency band are connected with the mechanisms of the thermoregulation, fluctuations of the vascular tone, the renin activity, and the secretion of the leptin [14]. The similarity of the frequencies implies the participation of these mechanisms in the genesis of the VLF spectral component. There are evidences of the increase of the VLF activity in case of the central nervous system damage, anxiety, and depression disorders [15].

The studied quantitative features of spectral analysis are

(i) spectral power of the HF, LF, and VLF components,
(ii) total power of the spectrum—TP,
(iii) normalized values of the spectral components by the total power—HF_*n*_, LF_*n*_, and VLF_*n*__,_
  (10) $$\begin{array}{c} {HF}_{n}=\frac{HF}{TP},\\ {LF}_{n}=\frac{LF}{TP},\\ {VLF}_{n}=\frac{VLF}{TP}, \end{array}$$
(iv) the LF/HF ratio, also known as the autonomic balance exponent,
(v) IC, the index of centralization,
  (11) $$\begin{array}{c} IC=\frac{HF+LF}{VLF}, \end{array}$$
(vi) IAS, the index of the subcortical nervous centers activation,
  (12) $$\begin{array}{c} IAS=\frac{LF}{VLF}, \end{array}$$
(vii) HF_max_, the maximal power of the HF spectral components,
(viii) RF, the respiration frequency, frequency that corresponds to the HF_max_ [16].

#### 2.2.4. Wavelet Transform

For nonstationary time series, one can also use the wavelet transform (wt), to simultaneously study time-frequency patterns. The general equation for continuous wavelet transform is as follows:

(13) $$\begin{array}{c} W\left(a,b\right)=\frac{1}{\sqrt{a}}\int s\left(t\right)\cdot \psi \left(\frac{t-b}{a}\right)dt, \end{array}$$

where *a* is the scale, *b* is the shift, *ψ* is the wavelet basis, and *s*(*t*) is the analyzed signal [17].

Moreover, the connection between the scale and the analyzed frequency is in accordance with the following:

(14) $$\begin{array}{c} a=\frac{f_{c}*f_{s}}{f}, \end{array}$$

where *f*_c_ is the central frequency of the wavelet basis, called by the *centfrq* function, *f*_s_ is the sampling frequency of the analyzed signal, and *f* is the analyzed frequency. For wavelet transform computation in this work, we used wavelet Coiflet of the fifth order [18].

It is possible to acquire same spectral features by means of the wavelet transform:

- Spectral power of the HF, LF, and VLF components
- Normalized values of the spectral components by the total power—HF_*n*_, LF_*n*_, and VLF_*n*_
- The LF/HF ratio.

Additionally, standard deviations SDHF(wt), SDLF(wt), and SDVLF(wt) of the HF_wt_(*t*), LF_wt_(*t*), and VLF_wt_(*t*) time series were tested as features. HF_wt_(*t*), LF_wt_(*t*), and VLF_wt_(*t*) are time series of the HF, LF, and VLF spectral components, respectively, acquired by means of the wavelet transform.

**Figure 2:**
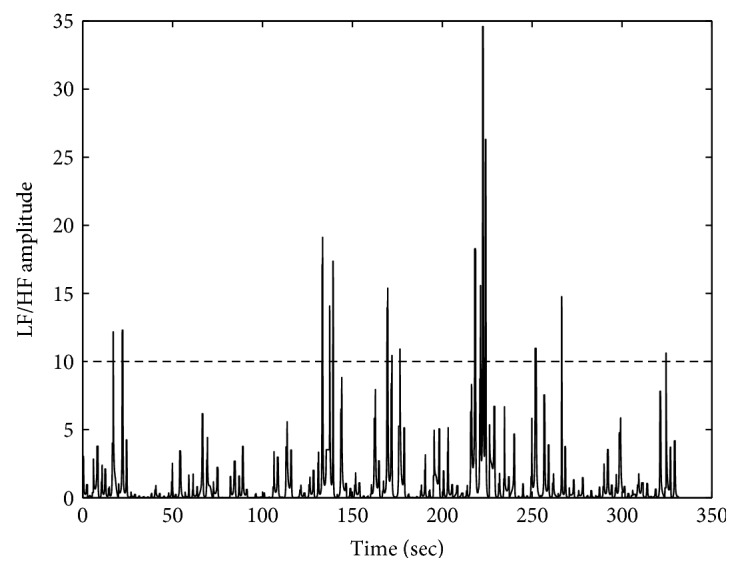
Example of *F*[LF_wt_/HF_wt_(*t*)] with the decision threshold.

Moreover, one can study informational characteristics of the wavelet transform by analyzing the *F*[LF_wt_/HF_wt_(*t*)] function (Figure 2). *F*[LF_wt_/HF_wt_(*t*)] is the continuous function of the LF/HF ratio. This function was not a smooth morphology. Its “excursions” (local dysfunctions) varies in case of functional loads, as the features of *F*[LF_wt_/HF_wt_(*t*)] is possible to use the number of dysfunctions Nd, the maximal value of dysfunction (LF/HF)_max_, and the intensity of dysfunction (LF/HF)_int_. By the dysfunction, we consider values of function that suppress decision threshold ∆ according to previous studies of our research group ∆ = 10 [19].

#### 2.2.5. Nonlinear Feature

As the nonlinear feature in this study, we have used the Hurst exponent calculated by the aggregated variance method. The variance can be written as follows:

(15) $$\begin{array}{c} Var\left|{\left(X\left(t_{2}\right)-X\left(t_{1}\right)\right)}^{2}\right|=\sigma^{2}{\left|t_{2}-t_{1}\right|}^{2H}, \end{array}$$

where *H* is the Hurst exponent and *X* is a time-series vector.

*H* can be defined as the slope exponent in the following equation:

(16) $$\begin{array}{c} \log\sigma_{rms}\left(\Delta X\right)=c+H\log\left|s\right|, \end{array}$$

where *σ*_rms_(∆*X*) is the standard deviation of the Δ*X* increments, corresponding to the time period *s*, and *с* is a constant [20].

Note that *H* > 0.5 corresponds to the process with trend, so-called persistent process, contrary *H* < 0.5 corresponds to antipersistent processes that have a tendency for trend change, and *H* = 0.5 is the random process [21].

#### 2.2.6. Multifractal Features

As nonlinear methods, we adopted the multifractal detrended fluctuation analysis (MFDFA) [22]. The algorithm and application features of the MFDFA method to estimation of short-term time series are described in details in [23].

The main steps of the method include the following:

- The detrending procedure with second degree polynomial on nonoverlapping segments where the length of the segments corresponds to the studied time scale boundaries.

In current study, we investigated time scale boundaries that correspond to the LF and VLF frequency bands: 6–25 sec and 25–300 sec, respectively. In our earlier works and by other authors, it was noted that multifractal analysis of the HF component is not informative because of the noising [24].

(ii) Determination of the fluctuation functions for *q* in range *q* = [−5, 5]:
  (17) $$\begin{array}{c} F_{x}\left(q,s\right)={\left\{\frac{1}{N_{s}}\overset{N_{s}}{\underset{v=1}{\sum }}{\left[\frac{1}{s}\overset{s}{\underset{k=1}{\sum }}{\left[NN\left(k\right)-{NN}_{v}\left(k\right)\right]}^{2}\right]}^{q/2}\right\}}^{1/q}, \end{array}$$
  where NN_*v*_ is the local trend in the segment ν, *N*_*s*_ is the number of segments, and *s* is the scale.
(iii) Estimation of the slope exponent *H*_*x*_ in log-log plot of the fluctuation function against scale *s* for each *q*:
  (18) $$\begin{array}{c} F_{x\left(q,s\right)}\approx s^{H_{x}\left(q\right)}. \end{array}$$
(iv) Calculation of the scaling exponent *τ*(*q*):
  (19) $$\begin{array}{c} \tau \left(q\right)=q\cdot H_{x}\left(q\right)-1. \end{array}$$
(v) The Legendre transform application for the probability distribution of the spectrum estimation:
  (20) $$\begin{array}{c} D\left(\alpha \right)=q\cdot \alpha -\tau . \end{array}$$

**Figure 3:**
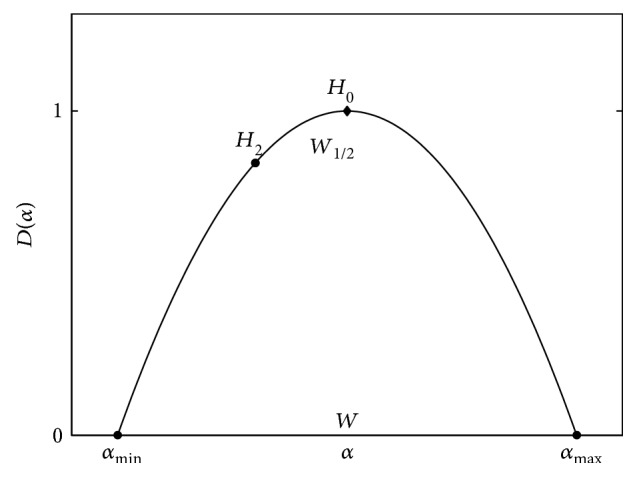
The features of multifractal analysis.

Figure 3 represents the main features of the multifractal spectrum estimated by the MFDFA method. Here, *H*_0_ is the height of the spectrum and represents the most probable fluctuations in the investigated time scale boundary of the signal; *H*_2_ is the generalized Hurst exponent (also known as correlation degree); *α*min represents behavior of the smallest fluctuations in the spectrum; *α*max represents behavior of the greatest fluctuations in the spectrum; and *W* = *α*_max_ − *α*_min_ is the width of multifractal spectrum that shows the variability of fluctuations in the spectrum. Multifractal characteristics are quantitative measures of the self-similarity and may characterize functional changes in the regulatory processes of the organism. In addition, we also tested the so-called 1/2-width measure of the spectrum, which is defined as *W*_1/2_ = ∣*H*_2_ − *H*_0_∣ [25].  Table 1 presents summary of all features used in this study.

### 2.3. Machine Learning Approaches

For the machine learning evaluation, the respective functions of the *sklearn* library were used [26]. The current paper describes supervised machine learning methods used. At first, the classifiers are trained using a training dataset. For that dataset, the class labels are known. After that, the efficacy of the classification is evaluated using test set of data. The efficacy is evaluated by comparing true labels of test set with those predicted by the model.

#### 2.3.1. Discriminant Analysis (DA)

In this work, two variants of the discriminant analysis were tested—linear and quadratic discriminant analyses (LDA and QDA). The LDA aims to find the best linear combination of the input features to properly separate studied classes. In the case of the QDA, the studied classes are separated by a quadratic function [27].

#### 2.3.2. *k*-Nearest Neighbors (*k*NN)

The *k*-nearest neighbors is one of the nonparametric machine learning approaches. In order to predict the class of the object, method chose the class, which is the most common among *k* “neighbors” of the object. Examples of the “neighbors” are picked from the training dataset. In the present study, different values of the *k* are tested—3, 4, and 5 [28].

#### 2.3.3. Support Vector Machine (SVM)

The base idea of the support vector machine methods is creation of the decision hyperplane which would separate different classes. In that case, the margin between two nearest points on the different sides of the hyperplane is maximal. In present study, the radial basis function (RBF) is used. For implementation in *python*, one have to specify the following: SVC(gamma = 2, C = 1) [29].

#### 2.3.4. Decision Trees (DT)

The decision trees classification model is built around a sequence of the Boolean queries. The sequence of such queries forms the “trees” structure. In the present work, variations of the classifier were analyzed—with fixed value of the maximal tree depth (*max_depth* = 5). The maximal depth feature points the maximal number of queries that is allowed to use before reaching leaf. The leaf node is the node that has no “children” [30].

#### 2.3.5. Naive Bayes (NB)

This method is based on the application of the Bayes' theorem with assumptions that data has strong (or naive) independence. In current study, the Gaussian distribution of data is assumed [31].

### 2.4. Semioptimal Search of the Noncorrelated Feature Space

#### 2.4.1. Feature Set Selection

**Figure 4:**
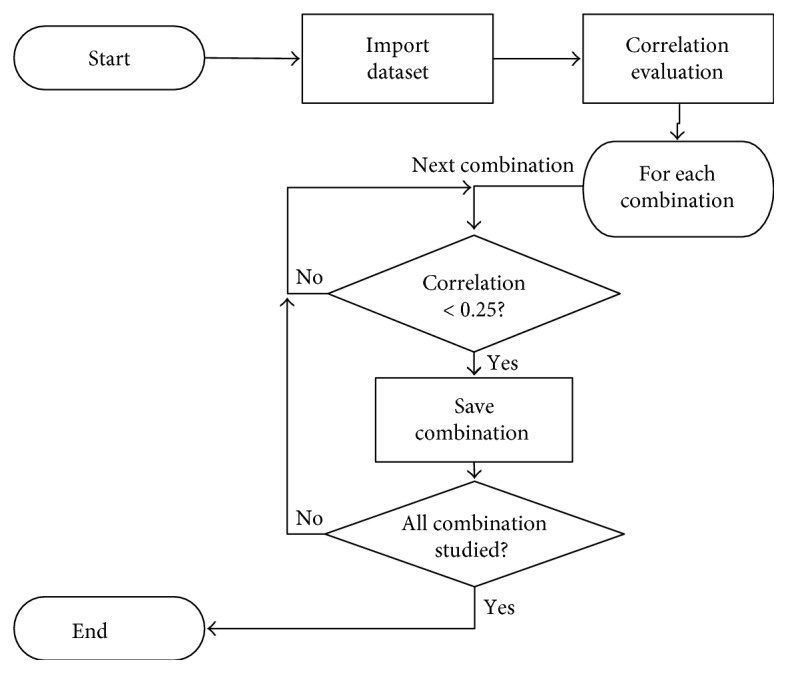
Flowchart of the noncorrelated combination selection.

In the current investigation, all possible combinations of all features were analyzed. However, it is well known that using combined correlated features in machine learning may lead to misleading results. Therefore, the first step in this investigation is to sort uncorrelated combinations. For this task, we compute the correlation coefficient. The whole flowchart of the script for noncorrelated feature combination selection is presented in Figure 4.

The threshold correlation value was set to 0.25. Usually, correlation more than 0.75 is considered to be high. Therefore, a value lower than 0.25 is a good benchmark for low correlation. In the current work, two to five feature combinations were made. In case of more than two feature combinations, the correlation was checked pairwise. When all calculation was finished, the noncorrelated features were saved to a file for future purposes.

Table 2 presents the total number of *n*-combinations for 53 features in case of *n* = [2, 3, 4, 5] and number of selected noncorrelated combinations. Table 2 shows that application of such selection leads to both, more appropriate results and significant reduction of the analyzed combinations set.

#### 2.4.2. Cross-Validation

**Figure 5:**
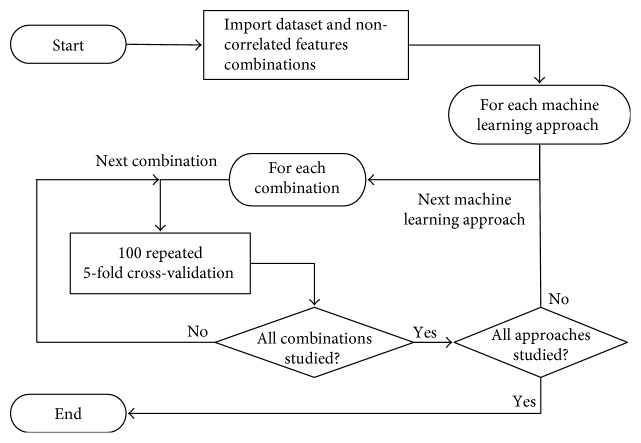
Flowchart of classifier efficacy evaluation algorithm.

Figure 5 presents a complete flowchart of the implemented algorithm for classifier efficacy evaluation.

Cross-validation implies division of the original datasets into *m* subsets, when *m*-1 subsets are used for the classifier training. The remaining part is used for the classifier test. The procedure is repeated *m* times. Such approach allows one to use dataset evenly [32].

In the current investigation, the number of random folds l was set to be 5. For the implementation of 5-fold cross-validation, we randomly divide the original dataset into 5 subsets. The division is implemented for both groups simultaneously. As the result, each subset included 6 healthy volunteers and 8 patients diagnosed with hypertension.

Many machine learning methods are sensitive to train set selection, so, in order to remove such influence, the cross-validation procedure was repeated 100 times with different folds. The repeated cross-validation allows to increase number of classification accuracy estimates [33].

Table 3 presents calculation times spent for each machine learning approach for different number of features in combinations. Calculation times are presented for all noncorrelated combinations. In accordance with Table 3, the fastest approach is the decision trees. The *k*-nearest neighbors approach is the slowest one.

## 3. Results

**Figure 6:**
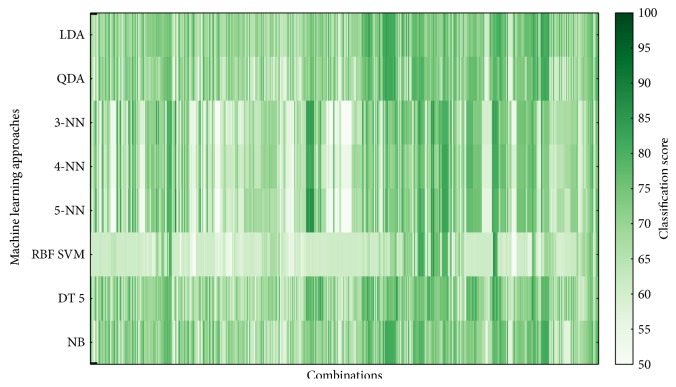
Classifier score for 2-feature combinations.

**Figure 7:**
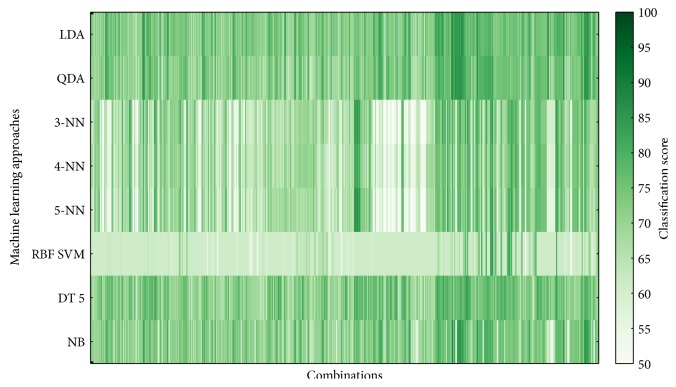
Classifier score for 3-feature combinations.

**Figure 8:**
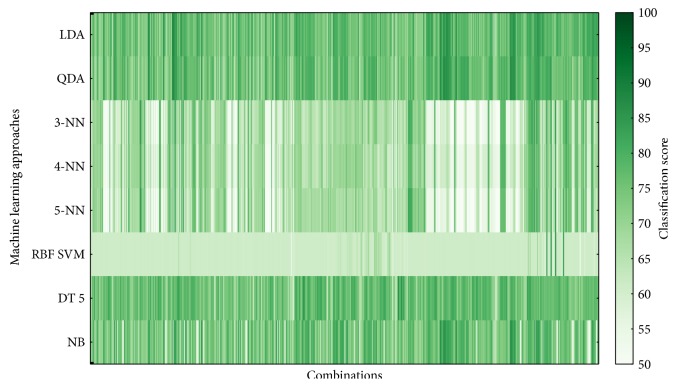
Classifier score for 4-feature combinations.

**Figure 9:**
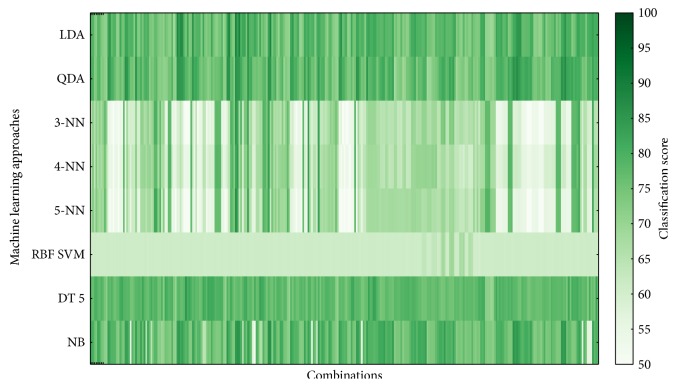
Classifier score for 5-feature combinations.

The classifier performance was averaged over 5 cross-validations and over 100 implementations. Figures 6–9 show overview of the classifier performance for all combinations for different numbers of features in combination. All color bars on Figures 5–8 have the same range—from 50 to 100%.

**Figure 10:**
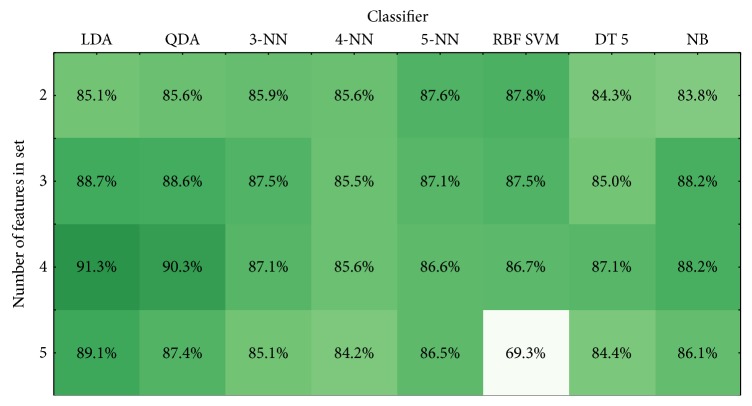
Maximal scores achieved by each learning machine approach.

Figure 10 presents maximal accuracy achieved by each classifier for different number of features in set.

According to the data presented in Figures 5–9, the highest classification is achieved by the discriminant analysis. Moreover, in Figures 5–8, it can be clearly seen that approaches of discriminant analysis have more combinations with relatively high score than any other approach. Furthermore, for the support vector machine approach, only few combinations have acceptable classification score level.

It is worthy to mention that generally classification accuracy rises as the number of features in the feature set increases. For 4-feature sets, the maximum is achieved—accuracy for 5-feature sets is lower for all machine learning approaches. It drops significantly in case of support vector machine approach.

Table 4 presents best results achieved by all machine learning approaches for 4-feature set.

Data in Table 4 shows that linear and quadratic DA not only achieve higher classification score but also have better stability of the results. Naïve Bayes classifier also has relatively high classification score and low deviation.

Among 53 studied features, 36 form combinations that have the classification score higher than 85.  Table 5 presents occurrences of the features among the combinations. The highest occurrences are noted for different spectral features, associated with VLF spectral band, LF/HF ratio, and statistical feature heart rate.

Table 6 presents 7 features that form combinations with accuracy higher than 90%. All these combinations consist of heart rate, one feature associated with LF/HF ratio, and two features associated with VLF spectral band.

## 4. Discussion

For discussion purposes, a comparison of the results of the current study with results of one of the commonly used procedure, principal components analysis (PCA), was executed. The PCA is a statistical procedure used to reveal the internal structure of the dataset [34]. In our case, features of different amplitude are used; PCA is known to be sensitive to the relative scaling of the feature dataset. Therefore, prior to the PCA application, the standardization procedure was implemented for each of 53 different features—subtraction of the mean value and after that division by the standard deviation.

Table 7 presents explained variance as well as the cumulative variance for the first 15 principal components. First 10 principal components explain 93% of the variance. Consequent principal components add 1% of the variance or less.

**Figure 11:**
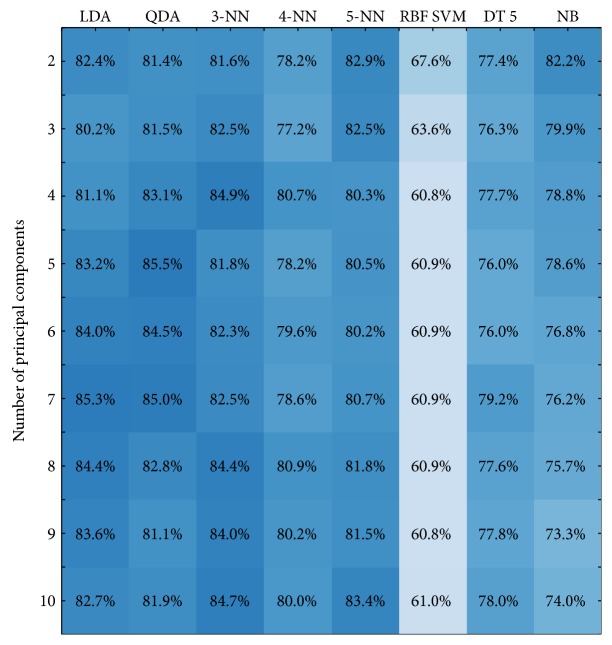
Scores of the PCA achieved by each learning machine approach.

In order to compare results of the semioptimal search of the noncorrelated feature space with PCA, combinations of the first 10 components were consequently tested for all machine learning approaches using 100 repeated 5-fold cross-validation.  Figure 11 presents the maximal results of classification accuracy achieved by each machine learning approach using combinations of the principal components.

Comparing the results of Figures 10 and 11, one can note that features found by the semioptimal search of the noncorrelated feature space reach higher classification accuracies than combinations of the principal components for all tested machine learning approaches.

## 5. Conclusions

In this work, various machine learning approaches were tested in task of the arterial hypertension diagnostics. In earlier works, the same datasets were used for investigation of the linear and quadratic DA methods [11]. The present work implies comparison of the DA methods with other machine learning approaches, like support vector machine, *k*-nearest neighbors, Naive Bayes, and decision trees.

The results of the current investigation showed that for the studied task, the application of the discriminant analysis (linear and quadratic) revealed to be the most appropriate classifiers. These approaches have high classification score and low deviations over different realizations. A set of four features in combination seems to be the optimal number, as the classification accuracy score is higher and more consistent than those for two, three, and five features in combination.

Prevalence of the VLF and LF/HF spectral features among best combinations might indicate that sympathetic nervous system takes an important part in the initialization of the arterial hypertension and maintenance of the increased vascular tone as well as increased cardiac output. These results are in accordance with scientists' interpretation of the arterial hypertension development [35, 36].

The results of the suggested approach were compared with data set prepared by the commonly used procedure of principal component analysis. Results of the *n*-feature noncorrelated sets have achieved higher classification accuracies than ones based on the dataset of the selected principal components.

In future works, our research group will continue to improve results on this problem. One of the investigations that are planned is to analyze robustness of the classifiers based on multiple signals recorded simultaneously. Among the other perspective directions of future investigation is usage of the advanced neural networks [37] and genetic algorithms [38] for feature extraction and classification.

## Figures and Tables

**Table 1:** List of studied features.

Feature	Description	Equation
*M*	Mean value of the R-R	(1)
HR	Heart rate	(2)
SDNN	Standard deviation of the R-R	(3)
CV	Coefficient of the variation	(4)
RMSSD	Square root of mean of squares of differences between successive R-R	(5)
NN50	Variation higher than 50 ms in R-R signal	—
*M*_0_	Mode of the R-R signal	—
VR	Variation range of the R-R signal	—
AM_0_	Amplitude of the mode	—
SI	Stress index	(6)
IAB	Index of autonomic balance	(7)
ARI	Autonomic rhythm index	(8)
IARP	Index of adequate regulation processes	(9)
HF(Fr)	High frequency Fourier spectral power	—
LF(Fr)	Low frequency Fourier spectral power	—
VLF(Fr)	Very low frequency Fourier spectral power	—
TP(Fr)	Total power of the Fourier spectrum	—
LF/HF(Fr)	Autonomic balance exponent of the Fourier spectrum	—
HF_max_(Fr)	Maximum power of the HF	—
HF_*n*_(Fr), LF_*n*_(Fr), and VLF_*n*_(Fr)	Normalized power of the HF, LF, and VLF Fourier spectrum	(10)
IC	Index of centralization	(11)
IAS	Index of the subcortical nervous center's activation	(12)
RF	Respiration frequency	—
HF(wt)	High frequency wavelet spectral power	—
LF(wt)	Low frequency wavelet spectral power	—
VLF(wt)	Very low frequency wavelet spectral power	—
HF_*n*_(wt), LF_*n*_(wt), and VLF_*n*_(wt)	Normalized power of the HF, LF, and VLF wavelet spectrum	—
SDHF(wt), SDLF(wt), and SDVLF(wt)	Standard deviations of the HF(*t*), LF(*t*), and VLF(*t*) wavelet time series	—
TP(wt)	Total power of the wavelet spectrum	—
LF/HF(wt)	Autonomic balance exponent of the wavelet spectrum	—
(LF/HF)_max_	Maximal value of dysfunctions	—
(LF/HF)_int_	Intensity of dysfunctions	—
Nd	Number of dysfunctions	—
*H*	Hurst exponent	(13)
*α*_min_ LF, *α*_min_ VLF	Smallest fluctuations of the LF and VLF spectral band	—
*α*_max_ LF, *α*_max_ VLF	Greatest fluctuations of the LF and VLF spectral band	—
WLF, WVLF	Spectrum width of the LF and VLF spectral band	—
*H*_2_LF, *H*_2_VLF	Correlation degree of the LF and VLF spectral band	—
*H*_0_LF, *H*_0_VLF	Spectrum height of the LF and VLF spectral band	—
*W*_1/2_LF, *W*_1/2_VLF	1/2-width measure of the LF and VLF spectral band	—

**Table 2:** Noncorrelated combination selection data.

*n*	Total *n*-combination number	Selected *n*-combinations	Calculation time, sec
2	1378	586	0.027
3	23,426	1669	0.477
4	292,825	1339	11.559
5	2,869,685	295	228.267

**Table 3:** Calculation times of classifier efficacy evaluation, sec.

Features in combinations	LDA	QDA	NN3	NN4	NN5	RBF SVM	DT	Naive Bayes
2	165	113	281	281	281	139	89	130
3	482	346	917	860	806	403	269	375
4	397	288	640	643	642	325	222	300
5	88	64	141	140	140	71	50	66

**Table 4:** Best classification scores.

Score, %	Features			
Linear discriminant analysis				
91.33 ± 1.75	HR	VLF_*n*_(Fr)	LF/HF(Fr)	VLF(wt)
90.30 ± 1.37	HR	VLF_*n*_(Fr)	VLF(wt)	(LF/HF)_int_
90.04 ± 1.85	HR	LF/HF(Fr)	VLF(wt)	VLFn(wt)
90.44 ± 1.60	HR	VLF_*n*_(Fr)	LF/HF(Fr)	SDVLF
90.11 ± 1.80	HR	LF/HF(Fr)	SDVLF	VLF_*n*_(wt)
90.16 ± 1.61	HR	SDVLF	VLF_*n*_(wt)	(LF/HF)_int_

Quadratic discriminant analysis				
90.31 ± 1.71	HR	VLF_*n*_(Fr)	LF/HF(Fr)	VLF(wt)

3-nearest neighbors				
87.14 ± 2.12	LF/HF(Fr)	SDVLF	VLF_*n*_(wt)	W_1/2_VLF

4-nearest neighbors				
85.56 ± 2.40	SDVLF	VLF_*n*_(wt)	LF/HF(wt)	W_1/2_VLF

5-nearest neighbors				
86.63 ± 1.30	HR	HF(Fr)	LF_*n*_(Fr)	W_1/2_VLF

Support vector machine, radial base function				
86.73 ± 2.24	IAS	RF	*a*_2_LF	WVLF

Decision trees, max depth 5				
87.10 ± 3.40	IARP	LF/HF(Fr)	IAS	WLF

Decision trees, no max depth				
87.34 ± 3.08	IARP	LF/HF(Fr)	IAS	WLF

Naïve Bayes classifier				
88.17 ± 1.07	VLF(Fr)	VLF_*n*_(Fr)	LF/HF(Fr)	W_1/2_LF

**Table 5:** Features occurrences for classification score higher than 85%.

Features	Occurrences, %	Features	Occurrences, %
VLF_*n*_(Fr)	50.89	Nd	4.73
VLF(Fr)	50.89	*M*_0_	4.73
VLF_*n*_(wt)	47.93	WLF	4.73
*W*_1/2_VLF	34.91	IARP	3.55
LF/HF(Fr)	34.32	IC	2.96
HR	33.73	HF(Fr)	2.96
SDVLF	30.18	*α*_2_ LF	2.96
(LF/HF)_max_	24.26	LF_*n*_(wt)	2.37
LF/HF(wt)	18.34	SI	2.37
(LF/HF)_int_	18.34	LF_*n*_(Fr)	1.78
*W*_1/2_LF	17.75	*α*_min_ LF	1.78
*H*	13.61	ARI	1.78
WVLF	13.02	HF_*n*_(Fr)	1.78
IAS	10.65	SDHF	1.18
VLF(wt)	7.69	IAB	0.59
RF	6.51	NN50	0.59
*α*_max_ LF	5.92	*α*_0_ LF	0.59
*M*	5.33	*α*_max_ VLF	0.59

**Table 6:** Feature occurrences for classification score higher than 90%.

Features	Occurrences, %
HR	100.00
LF/HF(Fr)	62.50
VLF(wt)	62.50
VLF_*n*_(wt)	50.00
VLF_*n*_(Fr)	50.00
SDVLF	37.50
(LF/HF)_int_	37.50

**Table 7:** Dataset analysis by PCA.

Principal component	Explained variance, %	Cumulative variance, %
1	34.88	34.88
2	17.65	52.54
3	13.03	65.57
4	8.87	74.45
5	5.38	79.83
6	4.10	83.93
7	3.55	87.47
8	2.42	89.89
9	1.78	91.67
10	1.36	93.03
11	1.01	94.04
12	0.95	94.98
13	0.84	95.82
14	0.74	96.56
15	0.61	97.17
